# Dynamic Tumor Tracking (DTT) for Hepatocellular Carcinoma Using the Vero4DRT Gimbaled Linac Stereotactic Body Radiation Therapy (SBRT) System

**DOI:** 10.3390/cancers17172926

**Published:** 2025-09-06

**Authors:** Ronan L. McDermott, Emma M. Dunne, Lok In Josephine Ma, Alanah M Bergman, Marie-Laure A. Camborde, Tania Karan, Ante Mestrovic, Emilie E. Carpentier, Mitchell C. C. Liu, Devin Schellenberg, Roy M. K. Ma

**Affiliations:** 1 Department of Radiation Oncology, British Columbia Cancer Agency, Vancouver Centre, 600 West 10th Avenue, Vancouver, BC V5Z 4E6, Canada; ronan.mcdermott@hse.ie (R.L.M.);; 2 Faculty of Medicine, The University of British Columbia, 317-2194 Health Sciences Mall, Vancouver, BC V6T 1Z3, Canada; 3 Department of Medical Physics, British Columbia Cancer Agency, Vancouver Centre, 600 West 10th Avenue, Vancouver, BC V5Z 4E6, Canadatania.karan@bccancer.bc.ca (T.K.);; 4 Department of Radiation Oncology, British Columbia Cancer Agency, Surrey Centre, 13850 96 Avenue, Surrey, BC V3V 1Z2, Canada

**Keywords:** SBRT, SABR, dynamic tumor tracking, HCC

## Abstract

**Background/Objectives**: Stereotactic body radiation therapy (SBRT) is a therapeutic option for hepatocellular carcinoma (HCC). This study reviewed outcomes and toxicities of SBRT for HCC using a gimbal-mounted linear accelerator and real-time monitoring system. **Methods**: A single-institution, retrospective review of SBRT for HCC using DTT between January 2018 and December 2020 was undertaken. Endpoints included local control (LC) and overall survival (OS). **Results**: A total of 74 patients with 82 tumors treated were identified. Median follow-up was 40.8 months. LC at 1, 3, and 5 years was 89.6%, 71.0%, and 59.9%, respectively. Median time to local failure was not reached. Median OS was 41.3 months (95% CI 30.7–51.8 months). OS at 1, 3, and 5 years was 89.2%, 60.6%, and 33.9%, respectively. On UVA, GTV ≥ 30 cm^3^ (*p* = 0.038), and PTV ≥ 150 cm^3^ (*p* = 0.010) were associated with an absolute drop in platelet count by ≥50,000/mm^3^ within six weeks of SBRT, while prior focal liver treatment (*p* = 0.097) showed a trend toward significance. Underlying viral cirrhosis (*p* = 0.033), A6 or higher pre-SBRT Child–Pugh score (*p* = 0.010), and pre-SBRT platelet count <100,000/mm^3^ (*p* = 0.017) were significant for a rise in Child–Pugh score of 2 points or more, and the volume of liver-GTV <1000 cm^3^ (*p* = 0.093) approached significance. **Conclusions**: SBRT using DTT is an effective therapeutic option for selected patients with HCC, providing acceptable local control and toxicity.

## Simple Summary

Stereotactic body radiation therapy (SBRT) is a non-invasive radiation technique that enables the delivery of high doses of radiation with precision. It is being used with increasing frequency as a treatment option for patients with primary liver tumors, classified as hepatocellular carcinoma (HCC). Real-time dynamic tumor tracking SBRT (DTT) using the Vero4DRT (Brainlab AG, Germany) platform has the advantage of tracking the location of the liver tumor as it moves with the patient’s breathing motion. This allows for more accurate targeting of the lesion, minimizing damage to the surrounding healthy tissue. Herein we report the clinical outcomes of SBRT for HCC using the DTT system at our institution.

## 1. Introduction

Hepatocellular carcinoma (HCC) represents a significant global health burden and is the third leading cause of cancer-related death worldwide [1].

The inherent underlying liver dysfunction in those with HCC, in addition to the usually advanced stage at the time of diagnosis, adds complexity to management. A multidisciplinary approach to decision making is, therefore, crucial to ensure appropriate treatment selection, considering baseline liver function, underlying co-morbidities, and tumor burden [2].

Stereotactic body radiation therapy (SBRT) is becoming an established locoregional treatment in patients with underlying comorbidities that preclude surgery as a bridging treatment to transplantation or in cases where the tumor location or size within the liver prevents any other form of liver-directed therapy (LDT) [3,4,5,6,7,8,9,10,11,12,13,14,15]. This highly conformal technique allows for the delivery of high biologically effective doses of radiation with millimetric precision to the target while controlling the differential dose to adjacent normal tissue.

Controlling intrafractional respiratory motion is a critical component of SBRT treatment delivery. Real-time dynamic tumor tracking SBRT (DTT) using the Vero4DRT (Brainlab AG, Germany) platform is one such motion management option. This technique allows for a reduction of treatment margins, consequently decreasing dose to surrounding organs at risk (OARs) and normal liver without sacrificing tumor dose [16,17,18,19,20].

The premise of this study was to report the clinical outcomes of SBRT for HCC using DTT at our institution.

## 2. Materials and Methods

### 2.1. Patients

A single-institution, retrospective review of all patients with hepatocellular carcinoma (HCC) treated with SBRT using dynamic tumor tracking (DTT) between January 2018 (when DTT for liver tumors was first initiated in this center) and December 2020 was performed. Inclusion criteria comprised the following: diagnosis confirmed by pathologic assessment or by radiologic confirmation using the LI-RADS criteria on multiphasic computed tomography (CT) or dynamic contrast-enhanced magnetic resonance imaging (MRI) in the setting of cirrhosis or chronic hepatitis B/C [21]; Barcelona Clinic Liver Cancer (BCLC) stage 0, A, or B; ≤3 discrete tumors (up to 15 cm) providing that uninvolved liver is >700 cm^3^ (or 10 cm^3^ per kilogram of body weight, whichever is less); multiphasic CT or dynamic contrast-enhanced MRI of the liver within 8 weeks of radiation planning; Child–Pugh score of ≤7 (unless pending transplant); aspartate aminotransferase (AST) and/or alanine aminotransferase (ALT) <3 times the upper range of normal and creatinine <150 µmol/L; ECOG 0-2 and case discussion at multidisciplinary tumor conference. Patients were excluded if they had evidence of direct invasion to the bile duct(s) or adjacent organs; distant or extrahepatic nodal spread; previous upper abdominal radiation excluding SBRT and Yttrium radio-embolization; cytotoxic chemotherapy or targeted biological therapy within 14 days of planned SBRT; or any gastric, duodenal, or variceal bleed within 2 months of SBRT; received prescribed doses below 30 Gy in 5 fractions and SBRT delivered using internal treatment volume (ITV) in free breathing. A total of 74 patients were identified meeting the defined inclusion criteria for analysis (Table 1).

### 2.2. Radiation Therapy

All patients were treated per institutional protocol. For each patient, radiopaque fiducials were inserted into the hepatic parenchyma adjacent to the tumor by an experienced interventional radiologist under ultrasound (US) guidance. Typically, between three (3) to five (5) gold (or other high atomic number) ‘seed’-type (typically 1 mm × 5 mm) fiducials were placed in a 3-dimensional arrangement within approximately 1 cm of the tumor and within the normal liver parenchyma

A post-fiducial insertion contrast-enhanced breath-hold CT was subsequently acquired, affirming the fiducial position and detailing pre-treatment hepatic disease status. Radiotherapy simulation was then performed using four-dimensional CT (4DCT) and breath–hold–exhale CT with intravenous contrast. For this, each patient was immobilized using a vacuum bag in the supine position with arms elevated. CT images were obtained using slices with 1.25–2.5 mm thickness. Delineation of gross tumor volume (GTV) was assisted with co-registration of the arterial phase of the post-fiducial contrast-enhanced diagnostic CT. A 5 mm geometric expansion edited at anatomical boundaries was created for the clinical target volume (CTV). A 5 mm planning target volume (PTV) expansion was subsequently applied. Treatment was delivered using DTT.

DTT was introduced in the center in 2017, and treatment was delivered using the Vero4DRT platform (Brainlab AG, Munich, Germany). Prior to this, patients were treated using motion encompassing internal target volumes (ITV) or respiratory gating. Following this introduction, all patients were assessed for both DTT suitability and respiratory gating. DTT was the preferred motion management method if a valid motion prediction model could be generated by the treatment unit with no minimum motion limit restrictions. This is because DTT adapts to the variability of the patient’s breathing pattern, both during the daily treatment and on different days over the entire course of the treatment. Abdominal compression was not used and the patient was treated while free-breathing.

The Vero4DRT system performs DTT by panning and tilting the beam up to ±2.4° in two directions to follow a moving target in craniocaudal and lateral directions, reaching any point within ±4.2 cm in the plane at isocenter perpendicular to the beam [19,22]. At the time of installation (2017), there were only two commercially available radiotherapy systems that could offer DTT: BrainLab Vero4DRT and Accuray Cyberknife. Compared to the competing Cyberknife technology, the Vero4DRT had the advantage of offering VMAT deliveries (including non-coplanar arcs), CBCT imaging, gantry-integrated dual-KV imaging (to optimize patient imaging angles), and larger field sizes (up to 15 × 15 cm^2^). Since that time, in 2019, Accuray started to market the Radixact unit, which has the option of being DTT-capable. This is a technology that combines tomotherapy helical radiation delivery with the Cyberknife dynamic tumor tracking technology (Synchrony) [23,24].

Radiation therapy was administered using non-coplanar intensity-modulated radiotherapy (IMRT) beams using a 6MV beam. A total dose of 30–45 Gy in 3–5 fractions (Table 1) delivered on alternative days was prescribed, aiming to cover the PTV with V100% >95%. In cases where the tumor location was near a critical organ at risk, prescriptions and PTV coverage were modified according to the treating physician’s judgement, with priority given to normal tissue constraints (see Table 2 for the list of dose–volume constraints (DVCs)). All contours underwent peer review as per institutional quality assurance protocol, and approved treatment plans received independent physics quality assurance checks.

### 2.3. Outcomes of Interest, Follow-Up, and Censoring

The primary outcome was time to local failure (local control). Secondary outcomes were overall survival (OS), progression-free survival (PFS), and treatment-related toxicity. Local failure was defined as the time from completion of SBRT to radiological or histological evidence of tumor recurrence within the treated target. In cases of equivocality, the abnormality on the follow-up scan was co-registered with the radiotherapy plan, and if the recurrence occurred within the 50% isodose line, it was considered a local failure. Patients who developed a recurrence at a site outside of the treated area were not censored for local recurrence. Those that did not undergo a local failure were censored at the date of the last CT or MRI follow-up imaging. OS was defined as the time from completion of SBRT until death from any cause. For OS, patients not undergoing the event of death were censored at the date they were last known to be alive. PFS was defined as the time from SBRT completion to any site of disease progression. For PFS, patients not undergoing the event of any disease progression were censored at the date of their last CT or MRI follow-up imaging. Clinical examinations, as well as diagnostic CT or MRI, were performed every 3–6 months after SBRT. Bloodwork, including platelet count, international normalized ratio (INR), bilirubin, alkaline phosphatase (ALP), gamma-glutamyl transferase (GGT), aspartate aminotransferase (AST), alanine aminotransferase (ALT), and albumin were collected before and between one- and three-month intervals post-SBRT to evaluate any deleterious effects on liver profile. Acute clinical toxicity of any grade was evaluated per the Common Terminology Criteria for Adverse Events (CTCAE) v4.0, and changes in platelet count, INR, and liver profile were recorded as a function of radiation-induced liver disease between baseline and within 12 weeks following completion of SBRT. Time intervals were measured from completion of treatment until the date of progression, death, or last follow-up.

### 2.4. Statistics

Statistical analyses were carried out using IBM^®^ SPSS^®^ statistical software version 25. The Kaplan–Meier method was used to estimate rates of local, intrahepatic control, DFS, and OS. To assess for significance and estimate the magnitude of the effects of the prognostic factors for local control, a Cox proportional hazards model was used, and statistical significance was accepted for *p*-values of <0.05. In multivariate analysis, factors with a *p*-value <0.1 from the univariate analysis were placed into a backward stepwise procedure to search for independent predictive factors. Covariates evaluated in the Cox model included age, gender, cause of cirrhosis, pre-SBRT Child–Pugh score, prescribed biological equivalent dose (BED), any prior focal liver therapy, tumor diameter, GTV volume, PTV volume, pre-SBRT platelet count, and volume of liver-GTV.

To allow for a comparison of radiotherapy doses used, BED with α/β 10 (BED_10_) was calculated.

## 3. Results

### 3.1. Patients and Treatment

A total of 74 patients with 82 treated tumours were identified and analyzed from a single-institution database of liver SBRT treatments since dynamic tumor tracking was initiated in January 2018. Patient and tumor characteristics are outlined in Table 1. All patients were reviewed at the multidisciplinary tumor board prior to SBRT treatment. Each patient underwent radio-opaque fiducial insertion followed by multiphasic CT before radiotherapy planning. Median age at treatment was 67.9 years (range 36.4–91.0). Median time from HCC diagnosis until SBRT was 14.1 months (range 2.9–185.3). Median tumor diameter was 3.3 cm (range 1.1–10.1). Median GTV was 15.8 cm^3^ (range 2.7–241.9). Median PTV was 99.6 cm^3^ (range 15.1–563.2). Hepatitis C virus was the commonest etiology of HCC (45.9%). Other causes included hepatitis B virus (28.4%), non-alcoholic steatohepatitis (12.2%), alcoholic liver disease (9.5%), primary biliary cirrhosis (2.7%), and cryptogenic (1.4%). Pre-SBRT Child–Pugh score (CPS) was available for all patients. The majority of patients were CPS A5 (71.6%). The remainder were A6 (20.3%), B7 (4.1%), and B8 (4.1%). No patients with CPS higher than B8 were treated. The most common dose and fractionation schedule used was 45 Gy in 3 fractions (58.6%). Other regimens used included 45 Gy in 5 fractions (33.0%), 42.5 Gy in 5 fractions (1.2%), 35 Gy in 3 fractions (1.2%), 40 Gy in 5 fractions (2.4%), 35 Gy in 5 fractions (1.2%), and 30 Gy in 5 fractions (2.4%). A total of 66.2% of patients underwent prior liver-directed therapy in the form of liver resection, percutaneous ablation, laparoscopic or open ablation, transarterial chemoembolization, Yttrium-90 radioembolization, bland embolization, or previous liver SBRT.

### 3.2. Clinical Outcomes

**Figure 1:**
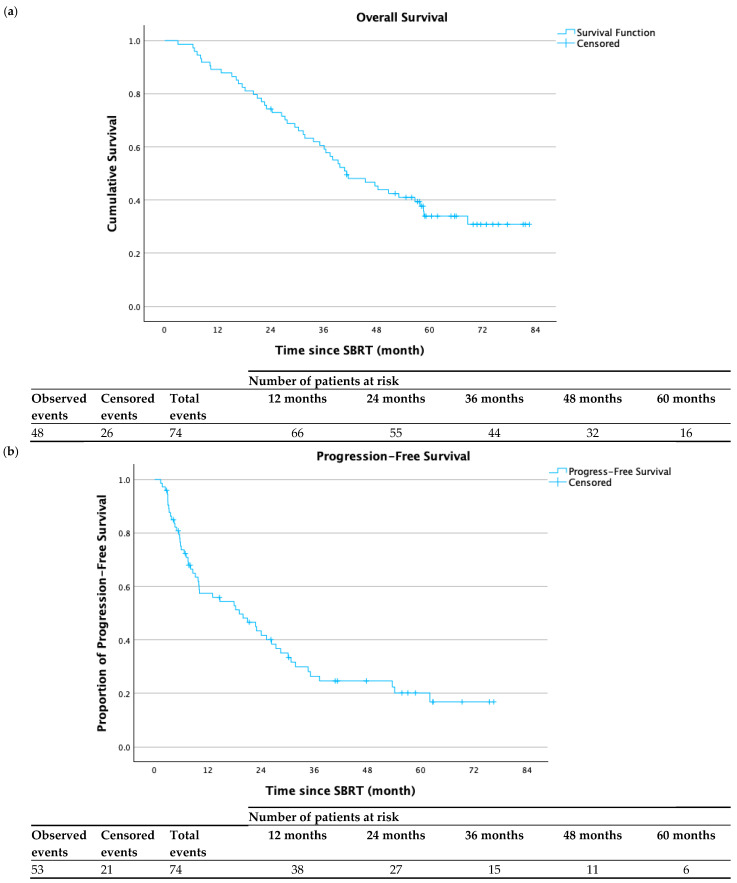

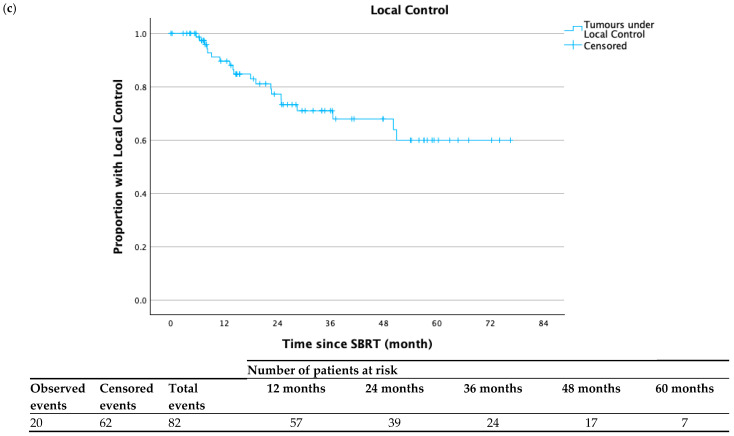
(**a**) Overall survival following completion of SBRT, with 48 observed events and 26 censored events. Median overall survival following completion of SBRT was 41.3 months (95% CI 30.7–51.8 months). (**b**) Progression-free survival following completion of SBRT, with 53 observed events and 21 censored events. Median progression-free survival was 19.1 months (95% CI 8.6–29.7 months). (**c**) Tumors under local control following completion of SBRT, with 20 observed events and 62 censored events. Median time to local failure was not reached.

The median overall follow-up for the entire cohort was 40.8 months. Median overall survival (OS) following completion of SBRT was 41.3 months (95% CI 30.7–51.8 months). OS at 1, 2, 3, and 5 years was 89.2%, 74.3%, 60.6%, and 33.9% respectively (Figure 1a). Fifty-three patients (71.6%) developed disease progression following SBRT completion. Median progression-free survival (PFS) was 19.1 months (95% CI 8.6–29.7 months). PFS at 1, 2, 3, and 5 years was 57.4%, 43.4%, 26.4%, and 20.2%, respectively (Figure 1b). Local control (LC) was assessed for the 82 lesions treated. Local failure occurred in 20 tumors (24.4%). Median time to local failure was not reached. LC of treated lesions at 1, 2, 3, and 5 years was 89.6%, 77.2%, 71.0%, and 59.9% respectively (Figure 1c). Twelve patients (16.2%) underwent orthotopic liver transplant after SBRT. Median time to transplant was 12.9 months (range: 2.1–50.1 months). Four of these patients had no residual tumor upon pathologic analysis. The remaining eight had a spectrum of therapeutic response in the treated area as described by the pathological report. Table 3 describes the individual tumor and dosimetric characteristics of the twelve patients who received a transplant.

### 3.3. Predictors of Outcome

In univariate analysis, female gender (HR 2.381, 95% CI 0.984–5.764; *p* = 0.054) was associated with, but did not reach, statistical significance for inferior local control, and underlying non-viral cause of cirrhosis (HR 3.138, 95% CI 1.276–7.719; *p* = 0.013) was statistically significant for inferior local control. Age at the time of treatment, ≥A6 Child–Pugh score pre-SBRT, prior liver-directed therapies, tumor diameter, GTV or PTV volume, dose-fractionation schedule, prescribed BED_10_, and CTV or PTV coverage (D99.5% BED_10_ < 100 Gy and/or BED_10_ < 80 Gy) were not significant for local control (Table 4).

In multivariate analysis, female gender (HR 1.781, 95% CI 0.690–4.599; *p* = 0.233) and underlying non-viral cause of cirrhosis (HR 2.564, 95% CI 0.976–6.733; *p* = 0.056) were not significant for inferior local control (Table 4).

### 3.4. Toxicity

For all patients, there was a median decline of platelets by 17,500/mm^3^ within 90 days post-treatment. There were seven patients (9.5%) who experienced an acute ≥ grade 3 platelet toxicity (defined as a total count of 25,000–50,000/mm^3^ post RT). All of these seven had underlying thrombocytopenia prior to starting SBRT.

When assessed for predictors of a fall in platelet count of 50,000/mm^3^ or more within six weeks after treatment, in univariate analysis, prior focal liver treatment (HR 0.391, 95% CI 0.129–1.186; *p* = 0.097), GTV ≥ 30 cm^3^ (HR 2.997, 95% CI 1.063–8.452; *p* = 0.038), and PTV ≥ 150 cm^3^ (HR 4.097, 95% CI 1.399–11.996; *p* = 0.010) were associated with a decrease in platelet count by ≥50,000/mm^3^ within six weeks of completing SBRT. Whereas age, gender, non-viral cause of cirrhosis, pre-SBRT Child–Pugh score (A6 or higher vs. A5), prescribed BED_10_, tumor diameter, pre-SBRT platelet count < 100,000/mm^3^, and volume of liver minus GTV < 1000 cm^3^ were not significant for a drop in platelet count of ≥50,000/mm^3^ post-SBRT. In multivariate analysis, PTV ≥ 150 cm^3^ (HR 4.290, 95% CI 0.858–21.443, *p* = 0.076) trended toward significance, whereas GTV ≥ 30 cm^3^ (HR 0.890, 95% CI 0.181–4.376, *p* = 0.886) and any prior focal liver treatment (HR 0.565, 95% CI 0.296–3.511, *p* = 0.628) did not show significance for a drop in platelet count of ≥50,000/mm^3^ post-SBRT (Table 5).

Eight patients (10.8%) had a deterioration in Child–Pugh score by ≥2 points. Table 6 described univariate and multivariate analyses of predictors of a rise in Child–Pugh score of 2 or more points. On univariate analysis, underlying non-viral cause of cirrhosis (HR 3.359, 95% CI 1.103–10.228; *p* = 0.033), A6 or higher pre-SBRT Child–Pugh score (HR 4.992, 95% CI 1.457–17.101; *p* = 0.010), pre-SBRT platelet count <100,000/mm^3^ (HR 4.335, 95% CI 1.306–14.384; *p* = 0.017) were significant for a rise in Child–Pugh score of 2 points or more. Volume of liver minus GTV of <1000 cm^3^ (HR 3.069, 95% CI 0.828–11.374; *p* = 0.093) and any prior focal liver treatments (HR 0.362, 95% CI 0.109–1.196; *p* = 0.096) trended toward significance. Age, gender, prescribed BED_10_, larger tumor diameter ≥3.3 cm, larger GTV ≥30 cm^3^, PTV ≥150 cm^3^ did not show significance for a rise in Child–Pugh score of 2 points or more. On multivariate analysis, pre-SBRT Child–Pugh score of A6 or more (HR 4.189, 95% CI 1.040–16.882; *p* = 0.044) remained significant and pre-SBRT platelet count <100,000/mm^3^ (HR 3.583, 95% CI 0.979–13.116; *p* = 0.054) trended toward significance whereas underlying non-viral etiology of cirrhosis (HR 1.452, 95% CI 0.388–5.428; *p* = 0.579) and volume of liver minus GTV of <1000 cm^3^ (HR 1.985, 95% CI 0.658–5.411; *p* = 0.210) did not retain significance for a rise in Child–Pugh score of 2 points or more.

## 4. Discussion

Our study has demonstrated that real-time dynamic tumor tracking SBRT (DTT) using the Vero4DRT is a safe and effective therapeutic option for selected patients with HCC.

Local control for the 82 lesions treated at 1, 3, and 5 years was 89.6%, 71.0%, and 59.9%, respectively, with the median time to local failure not yet reached. Non-viral cause of cirrhosis was statistically significant for inferior local control in univariate analysis (*p* = 0.013) and approached statistical significance in multivariate analysis (*p* = 0.056). It is suspected that virus-induced HCC is more radiosensitive than non-virus-induced HCC. A total of 74% of patients in this series had an underlying viral cause of HCC; this may account for the non-significance of prescribed BED_Gy10_, tumor diameter, volume of treatment, and PTV coverage for local control. Since the median progression-free survival was 19.1 months, this meant that most of the treated lesions were still well controlled with no viable tumor when a new HCC developed in the rest of the liver that required further treatment. With a median follow-up of 40.8 months, the overall survival at 1, 3, and 5 years was 89.2%, 60.6% and 33.9%, respectively, which is in keeping with several published series of HCC treated with SBRT. SBRT also compares favorably with other liver-directed therapies with similar overall survival as transarterial chemoembolization (TACE) and radiofrequency ablation (RFA) in unresectable disease [25,26,27].

Our results are in line with a recently reported systematic review and meta-analysis of outcomes of 1889 patients with HCC treated with SBRT across seventeen observational studies. They reported local control at 3 and 5 years of 84% (95% CI, 77–90%) and 82% (95% CI, 74–88%), respectively, and similar 3- and 5-year overall survival rates of 57% (95% CI, 47–66%) and 40% (95% CI, 29–51%), respectively. Prognostic factors of improved overall survival were size < 3 cm, Eastern region, Child–Pugh score < B8, and BCLC stage 0 and A. Tumor volume was the only prognostic factor for local control [28].

Our study demonstrated that SBRT using DTT was safe. Eight patients (10.8%) had a deterioration in their Child–Pugh score by ≥2 points within 3 months post-SBRT. In multivariate analysis, pre-SBRT Child–Pugh score of A6 or more was statistically significant (*p* = 0.044) for deterioration in their Child–Pugh score by ≥2 points within 3 months post-SBRT, whereas pre-SBRT platelet count <100,000/mm^3^ trended toward significance (*p* = 0.054). This is in keeping with the established poor prognostic value of these two factors for both HCC and liver cirrhosis

SBRT is being used with increasing frequency in patients with HCC in a multitude of settings. Some of the challenges with delivering high radiation doses to tumors of the liver include how to effectively manage respiratory motion, where movement due to respiration can be up to several centimetres in amplitude [29,30]. Such methods currently include treating relatively larger volumes in a motion encompassing (ITV) method, using breath–hold techniques or respiratory gating, applying abdominal compression, or through DTT. Using DTT means, an ITV margin is not required, and a 100% duty cycle for dose delivery can be achieved [31,32,33]. Initial work by our center when establishing VERO DTT demonstrated a 38% reduction in PTV volume using DTT compared to an ITV method [34]. This is of particular relevance in HCC, which is commonly accompanied by a diseased liver. Many of these patients typically have heavily pre-treated livers at the point of referral; in this study, 66.2% of patients underwent prior liver-directed therapy. Since most patients not eligible for a liver transplant will develop a new HCC in the future, liver function preservation post-SBRT is of paramount importance to maximize the options of future treatment.

Our institution developed a 4D dose calculation protocol for liver SBRT patients that accounts for their breathing motion during DTT treatments [35]. This protocol recalculates an optimized treatment plan on all 10 phases of the patient’s 4DCT, adjusting the beam’s angle appropriately on each phase to model the Vero4DRT pan/tilt angle during tracking. These 10 dose distributions can then be accumulated together using deformable image registration to produce a single 4D dose distribution accounting for the patient’s breathing motion. To the best of the author’s knowledge, this was the first study to model the Vero4DRT unique tracking geometry within the treatment planning system (TPS). This 4D dose calculation protocol was also implemented using Monte Carlo simulations to create a secondary verification system for the TPS 4D dose distribution calculations [36,37]. In addition, 4D treatment planning strategies were developed to account for the patient’s breathing motion during plan creation.

While studies report similar clinical outcomes to our own results herein, these data, presented in this retrospective review, must be taken in the context of several limitations and potential bias. This population is an extremely heterogeneous cohort of variation in underlying comorbidity, liver dysfunction, and tumor characteristics. Patient factors, tumor burden, and location, as well as underlying liver disease, influence the delivered dose, which, in turn, influences local control. Follow-up imaging and blood work analyses were performed at the discretion of the treating oncologist and not at predetermined intervals, such as those within a prospective clinical trial. Radiological reporting was not standardized across the cohort either. It is recognized that these inherent limitations may influence the reporting of local control and progression-free survival rates.

## 5. Conclusions

SBRT is an established and effective treatment for HCC used in several situations, from definitive management to salvage post other liver-directed therapies or as a bridge to transplantation. Delivery using DTT has many advantages and demonstrates high rates of durable local control, which can be seen in pathological analysis of transplanted livers. Sustained patterns of local failures still emphasize the need for dedicated long-term follow-up post-treatment.

## Figures and Tables

**Table 1:** Clinical characteristics of patients and tumors.

Parameter	Number (%)
**Patient Characteristics**	
Total patients	74
Total tumors treated	82
Median age at diagnosis—(range), y	65.3 (36.0–87.5)
Median age at treatment—(range), y	67.9 (36.4–91.0)
*Sex*	
Male	54 (73.0%)
Female	20 (27.0%)
*Cause of Cirrhosis*	
HCV	34 (45.9%)
HBV	21 (28.4%)
NASH	9 (12.2%)
ALD	7 (9.5%)
PBC	2 (2.7%)
Cryptogenic	1 (1.4%)
*Child–* *Pugh Score Pre-SBRT*	
A5	53 (71.6%)
A6	15 (20.3%)
B7	3 (4.1%)
B8	3 (4.1%)
*Prior Local Liver Treatment*	
Yes	49 (66.2%)
No	25 (33.8%)
**Tumor Characteristics**	
*Tumor Max. Diameter*	
Median (range), cm	3.3 (1.1–10.1)
*Gross Tumor Volume*	
Median (range), cm^3^	15.8 (0.7–241.9)
*Clinical Target Volume*	
Median (range), cm^3^	39.5 (4.2–303.4)
*Planning Target Volume*	
Median (range), cm^3^	99.6 (15.1–563.2)
*Prescribed Dose Fractionation (BED_Gy10_)*	
45 Gy, 3 fractions (112.5 Gy_10_)	48 (58.6%)
45 Gy, 5 fractions (85.5 Gy_10_)	27 (33.0%)
42.5 Gy, 5 fractions (78.6 Gy_10_)	1 (1.2%)
35 Gy, 3 fractions (75.8 Gy_10_)	1 (1.2%)
40 Gy, 5 fractions (72 Gy_10_)	2 (2.4%)
35 Gy, 5 fractions (59.5 Gy_10_)	1 (1.2%)
30 Gy, 5 fractions (48 Gy_10_)	2 (2.4%)

Abbreviations: HCV, hepatitis C; HBV, hepatitis B; NASH, non-alcoholic steatohepatitis; ALD, alcoholic liver disease; PBC, primary biliary cholangitis.

**Table 2:** Dose–volume constraints.

	3 Fractions	5 Fractions
**Spinal Cord PRV**	D_max_ ≤ 20.3 Gy	D_max_ ≤ 25.3 Gy
Liver minus GTV	≥700 cm^3^ ≤ 17 Gy	≥700 cm^3^ ≤ 21 Gy
Duodenum	D_max_ ≤ 22.2 Gy	D_max_ ≤ 32 Gy
Stomach	D_max_ ≤ 22.2 Gy	D_max_ ≤ 32 Gy
Small Bowel	D_max_ ≤ 25.2 Gy	D_max_ ≤ 29 Gy
Large Bowel	D_max_ ≤ 28.2 Gy	D_max_ ≤ 38 Gy
Heart	D_max_ ≤ 30 Gy V24 Gy ≤ 15 cm^3^	D_max_ ≤ 38 Gy V24 Gy ≤ 15 cm^3^
Esophagus	D_max_ ≤ 27 Gy	D_max_ ≤ 35 Gy
Chest Wall	D_max_ ≤ 44 Gy V30 Gy ≤ 30 cm^3^	D_max_ ≤ 55 Gy V37 Gy ≤ 30 cm^3^
Great Vessels	D_max_ ≤ 44 Gy	D_max_ ≤ 51.5 Gy

**Table 3:** Tumor and dosimetric characteristics of the patients who received orthotopic liver transplant (OLT) after SBRT.

Patient Number	GTV Volume (cm^3^)	Prescribed BED (Gy_10_)	CTV D99.5% (Gy10)	Time from SBRT to Transplant (Months)	OLT Pathology Report
1	13.2	112.5	135.8	15.7	No residual tumor
2	17.6	85.5	101.8	8.6	No residual tumor
3	9.2	85.5	91.3	2.1	30% necrosis
4	2.8	112.5	129.8	8.1	No residual tumor
5	3.1	112.5	130.6	50.1	Friable necrotic center with 2.2 cm viable tumor
6	31.4	112.5	121.5	10.1	Partial necrosis
7	5.7	85.5	75.2	5.9	No residual tumor
8	27.5	112.5	137.6	18.2	Viable tumor present
9	53.5	112.5	116.7	18.3	90% necrosis
10	50.2	72	58.5	6.3	30% necrosis
11	5.8	112.5	123.5	16.7	50% necrosis
12	13.2	85.5	94.6	39.2	Viable tumor present

**Table 4:** Univariate and multivariate analysis of tumor local control.

Variable	*p*-Value	HR (95% CI)	*p*-Value	HR (95% CI)
	Univariate (Cox Regression)		Multivariate (Cox Regression)	
Age at treatment	0.457	1.016 (0.974–1.061)		
Gender (female vs. male)	0.054	2.381 (0.984–5.764)	0.233	1.781 (0.690–4.599)
Cause of cirrhosis (non-viral vs. HBV/HCV)	0.013	3.138 (1.276–7.719)	0.056	2.564 (0.976–6.733)
Pre-SBRT CPS (A6 or higher vs. A5)	0.383	0.520 (0.120–2.258)		
Prescribed dose, fractionation (45 Gy, 3 vs. other doses, fractionations)	0.590	1.288 (0.513–3.231)		
Prescribed BED_10_ (<112.5 Gy)	0.590	0.777 (0.310–1.949)		
Prior focal liver treatment	0.700	0.835 (0.333–2.095)		
Tumor diameter (≥3.3 cm)	0.532	1.325 (0.548–3.205)		
GTV volume (≥30 cm^3^)	0.285	1.621 (0.669–3.925)		
CTV D99.5% BED_10_ <100 Gy	0.804	1.118 (0.462–2.704)		
PTV volume (≥150 cm^3^)	0.169	1.851 (0.770–4.453)		
PTV D99.5% BED_10_ <80 Gy	0.931	0.962 (0.398–2.326)		
PTV D99.5% BED_10_ <100 Gy	0.904	0.943 (0.361–2.459)		

**Table 5:** Univariate and multivariate analysis of a decrease in platelet count by 50,000/mm^3^ or more within 6 weeks of completing SBRT.

Variable	*p*-Value	HR (95% CI)	*p*-Value	HR (95% CI)
	Univariate (Cox Regression)		Multivariate (Cox Regression)	
Age at treatment	0.210	1.030 (0.983–1.080)		
Gender (female vs. male)	0.177	2.052 (0.722–5.829)		
Cause of cirrhosis (Non-viral vs. HBV/HCV)	0.283	1.898 (0.589–6.123)		
Pre-SBRT CPS (A6 or higher vs. A5)	0.956	0.964 (0.268–3.474)		
Prescribed BED_10_ (<112.5 Gy)	0.232	1.910 (0.661–5.522)		
Any prior focal liver treatment	0.097	0.391 (0.129–1.186)	0.628	0.565 (0.296–3.511)
Tumor diameter (≥3.3 cm)	0.101	2.470 (0.838–7.278)		
GTV volume (≥30 cm^3^)	0.038	2.997 (1.063–8.452)	0.886	0.890 (0.181–4.376)
PTV volume (≥150 cm^3^)	0.010	4.097 (1.399–11.996)	0.076	4.290 (0.858–21.443)
Pre-SBRT platelet count (<100,000/mm^3^)	0.105	0.022 (0.000–2.217)		
Volume of liver-GTV (<1000 cm^3^)	0.554	1.574 (0.351–7.050)		

**Table 6:** Univariate and multivariate analysis of an increase in Child–Pugh score by ≥2 points within 3 months of completing SBRT.

Variable	*p*-Value	HR (95% CI)	*p*-Value	HR (95% CI)
	Univariate (Cox Regression)		Multivariate (Cox Regression)	
Age at treatment	0.239	0.969 (0.921–1.021)		
Gender (female vs. male)	0.142	2.273 (0.759–6.806)		
Cause of cirrhosis (non-viral vs. HBV/HCV)	0.033	3.359 (1.103–10.228)	0.579	1.452 (0.388–5.428)
Pre-SBRT CPS (A6 or higher vs. A5)	0.010	4.992 (1.457–17.101)	0.044	4.189 (1.040–16.882)
Prescribed BED_10_ (<112.5 Gy)	0.267	1.926 (0.606–6.121)		
Any prior focal liver treatment	0.096	0.362 (0.109–1.196)		
Tumor diameter (≥3.3 cm)	0.521	1.433 (0.477–4.301)		
GTV volume (≥30 cm^3^)	0.265	1.873 (0.621–5.645)		
PTV (≥150 cm^3^)	0.612	1.337 (0.436–4.103)		
Pre-SBRT platelet count (<100,000/mm^3^)	0.017	4.335 (1.306–14.384)	0.054	3.583 (0.979–13.116)
Volume of liver-GTV (<1000 cm^3^)	0.093	3.069 (0.828–11.374)	0.210	1.985 (0.658–5.411)

## Data Availability

Research data are stored in an institutional repository and will be shared upon request to the corresponding author.
